# Session Introduction: TOWARDS ETHICAL BIOMEDICAL INFORMATICS: LEARNING FROM OLELO NOEAU, HAWAIIAN PROVERBS

**Published:** 2023

**Authors:** Peter Y. Washington, Noelani Puniwai, Martina Kamaka, Gamze Gürsoy, Nicholas Tatonetti, Steven E. Brenner, Dennis P. Wall

**Affiliations:** Department of Information & Computer Sciences, University of Hawaii at Manoa, Honolulu, HI 96822, USA; Kamakakuokalani, University of Hawaii at Manoa, Honolulu, HI 96822, USA; Department of Native Hawaiian Health, University of Hawaii at Manoa, Honolulu, HI 96822, USA; Department of Biomedical Informatics, Columbia University, New York, NY 10032, USA; Department of Biomedical Informatics, Columbia University, New York, NY 10032, USA; Department of Plant & Microbial Biology, University of California, Berkeley, Berkeley, CA 94720, USA; Department of Pediatrics (Systems Medicine), Biomedical Data Science, and Psychiatry & Behavioral Sciences, Stanford University, Stanford, CA, 94305, USA

**Keywords:** Ethics, Bioethics, Privacy, Fairness, Bias, Biomedical Data Science, Pono

## Abstract

Innovations in human-centered biomedical informatics are often developed with the eventual goal of real-world translation. While biomedical research questions are usually answered in terms of how a method performs in a particular context, we argue that it is equally important to consider and formally evaluate the ethical implications of informatics solutions. Several new research paradigms have arisen as a result of the consideration of ethical issues, including but not limited for privacy-preserving computation and fair machine learning. In the spirit of the Pacific Symposium on Biocomputing, we discuss broad and fundamental principles of ethical biomedical informatics in terms of Olelo Noeau, or Hawaiian proverbs and poetical sayings that capture Hawaiian values. While we emphasize issues related to privacy and fairness in particular, there are a multitude of facets to ethical biomedical informatics that can benefit from a critical analysis grounded in ethics.

## 1. Introduction

The field of biomedical informatics is intrinsically tied to ethics, as a large portion of innovations are developed for the explicit purpose of advancing human health. However, every innovation involves a wide array of stakeholders, such as clinicians, patients, family members of the patients, healthy individuals whose data are used to support an informatics solution, and many others. A solution that improves the health of one stakeholder may harm or put at risk another stakeholder in often inadvertent and subtle ways.

Considering the ethics of biomedical informatics solutions may lead to varying conclusions depending on the ethical framework used to conduct the analysis. Utilitarianism, for example, is a framework centered around doing the greatest amount of good for the largest number of people. Deontological ethics, by contrast, centers around doing the morally right action regardless of the number of people affected. One can propose countless examples of decisions that may align with one ethical theory but directly conflict with another. For example, collecting large swaths of training data that contain protected health information may be ideal from a utilitarian standpoint, as the model would be used to help a large number of people, but might be unethical from a deontological view without extensive privacy protections in place.

Here, we consider another ethical perspective: Olelo Noeau, or Native Hawaiian proverbs that capture Native Hawaiian values and the Hawaiian worldview. The Pacific Symposium on Biocomputing (PSB) takes place in Hawaii every year. As such, we center this introduction on a discussion of Native Hawaiian values as they relate to the field of biomedical informatics. While we acknowledge that many Native Hawaiian values have variety and layers to their meaning, for our purposes, we will focus on the more commonly understood meanings of these phrases. We summarize relevant Olelo Noeau for biomedical informatics in Table 1.

## 2. Ike aku, ike mai, kokua aku kokua mai; pela iho la ka nohona ohana. Family life requires an exchange of mutual help and recognition.

Ohana, the word for family, is one of the key Hawaiian principles that defines Hawaiian culture. The Hawaiian proverb “*Ike aku, ike mai, kokua aku, kokua mai; pela iho la ka nohona ohana*” literally describes the importance of a human-centered design process - “*recognize and be recognized, help and be helped; such is family life*” [1]. Native Hawaiian social structure is centered around extended families. For example, illnesses affect the entire Ohana because what impacts one impacts all. Laulima is also a pillar of Hawaiian culture: goals must be achieved by collaboration and cooperation. Traditionally, survival depended on this.

Following this ideal, one might suggest that biomedical informatics solutions should be developed with to work for all stakeholders, regardless of socioeconomic, demographic, political, or geographic factors. This includes involving all stakeholders in the development and design process, often with the aid of established human-centered design practices.

Digital solutions for various health conditions often and increasingly incorporate informatics solutions. For example, the SuperpowerGlass system developed by some of the authors at Stanford [2] was initially designed using in-person human-centered design sessions with participants. Before even the first quantitative feasibility study was conducted, iterative design sessions with participants were completed, and parent and child stakeholders were extensively interviewed by the study team [3–4]. Qualitative feedback was collected and coded to inform the updated design decisions of future iterations of the wearable therapeutic [5]. Only after these design sessions was the SuperpowerGlass system tested in feasibility studies [6–8] and a formal randomized controlled trial [9]. The process of co-designing with the end users of a medical solution can prevent situations where extensive time and effort is put into developing elaborate solutions that are ultimately disregarded by patients and clinicians as being unusable or unethical.

## 3. Ike i ke au nui me ke au iki. Know the big current and the little current.

The Hawaiian proverb “*Ike i ke au nui me ke au iki*” translates to “*know the big current and the little current*” in English, meaning that it is valuable to recognize the importance of all knowledge, be it small or large [1]. Ensuring the dialogue of data sources and data analysis is inclusive of all supports this ideal.

Similarly, the concept of Pono refers to the ideal balance of equity and abundance among all living and non-living entities [19]. A Pono concept is larger than the defense of right conduct that structures our conversations around ethics and ensures that our motivation in seeking pono is for the prosperity of all communities.

Fairness in machine learning is particularly important in the contexts of biology, medicine, and health. Machine learning models that make a diagnostic prediction, for example, can be problematic if the level of fidelity of the prediction of disease status is inconsistent across demographic groups. Machine learning classifiers are limited by the input data that are used to train them, and in many instances, the training data are unbalanced and biased. Due to differences in representation levels at the granularity of a hospital, city, or country, it may be impossible to collect balanced data sets without discarding large amounts of data from the majority class. Recent algorithmic techniques enable increased fairness, including data augmentation to upsample the underrepresented groups [10–12], enforcing a flavor of fairness in the loss function or otherwise imposing an algorithmic constraint [13–14], or post-processing methods for redefining the prediction thresholds for a black box model [15–17]. Some argue that beyond issues with data are fundamental biases in the quantitative methodologies themselves, which can put underserved populations at a disadvantage. Maggie Walter and Chris Andersen explore this topic in “Indigenous Statistics: A Quantitative Research Methodology” [18], discussing issues such as the inherent power dynamics between non-Indigenous and Indigenous populations in statistical and policy discourse and ways that data collection methods are designed to only collect data of certain types.

## 4. Kanukanu, huna i ka meheu, i ka maawe alanui o Kapuukolu. Covering with earth, hiding the footprints on the narrow trail of Kapuukolu.

This Hawaiian proverb shares a value of privacy and guarding of personal information from those who pry. “In ancient times a person who did not want to be traced by his footsteps carefully eradicated them as he went” [1]. While these ideals can extend to a variety of topics in biomedical informatics, we hone in on respect of the participants whose data are used to develop biomedical innovations. We discuss respect for privacy in particular, which is the greatest concern of participants who share their data.

The concept of Kapu similarly reflects the respect required of personal data and the privilege of working with information that can be identifiable [47]. Kapu references not only the interaction with the dataset, but the ability to safeguard, protect and honor that which comprises the sacredness and dignity of each individual.

Biomedical data are sensitive by definition, often containing protected health information and identifiable information. It is crucial to share these data with the broader community in order to advance scientific progress [20–21]. However, the potential for data breaches must be accounted for. In biomedical informatics, avenues for potential breaches extend beyond traditional hacking and computer security issues. Risks specific to this field include but are not limited to identifying the genome of a single individual from within a larger dataset [22–25], cross-referencing multiple databases using demographic and familial information [26–27], inherently identifying multimedia datasets [28–32], and performing diagnostic assessments with humans in the loop [33–38]. Other considerations are the management of very small data sets, since the careless release of these could compromise not only privacy, but also dignity of subjects. Current solutions to these issues include homomorphic encryption [39–41], running privacy audits through bioinformatics tools [42–43], data sanitization [44], and differential privacy [45], and federated learning [46].

## 5. He waiwai nui ka lokahi; Unity is a precious possession. (Lokahi as it relates to Balance and Harmony)

**Fig. 1.**
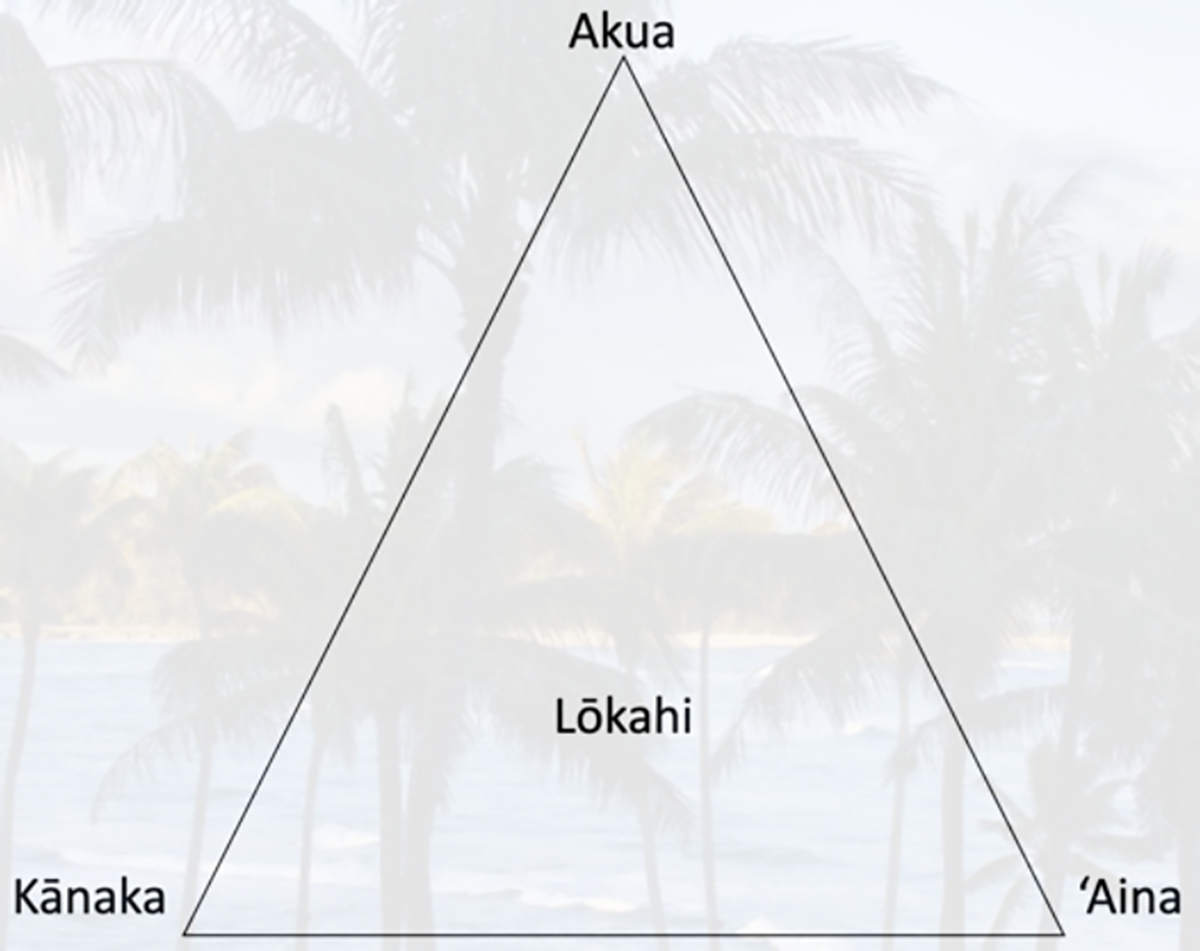
Lokahi triangle, consisting of spirituality (akua), humankind (Kanaka), and nature (Aina). Together, these elements represent balance.

Lokahi is the concept of balance; in the Native Hawaiian worldview it incorporates the balance between spirituality (Akua), humankind (Kanaka), and nature (Aina). These three pillars of Lokahi are embodied in the Lokahi triangle (Figure 1). The values of the Lokahi triangle are central to the Hawaiian notion of holistic health, including in contemporary health practices in Hawaii [48]. Lokahi is encompassed in the Hawaiian proverb “*He waiwai nui ka lokahi*”, or “*unity is a precious possession*” [1]. Lokahi translates directly to ethical biomedical informatics as the marriage of traditional performance metrics (such as accuracy, mean squared error, F1-score, and AUROC) with metrics that contain an ethical component (such as attack success rate for privacy and demographic parity for fairness). Often, these metrics can be at direct odds with each other. For example, it has been repeatedly documented that improving fairness can often detriment model performance and vice versa [49–55]. Considering our framework perspective, consideration for what is ultimately the best solution for this concept is the one that does the pono (proper) thing and finds a way to balance both.

## 6. Closing Thoughts

We emphasize that the Hawaiian cultural concepts are not simply words/phrases but ways of living. Biomedical informatics is a discipline that is inherently human-centered, and yet the quantitative logistics of the field can stray far from this central core, resulting in researchers forgetting the ethical implications of their work. We hope that this short piece will inspire PSB attendees to become Alakai, or leaders, in the incorporation of values-driven perspective in all facets of biomedical informatics research. Doing so could help avoid ethical complications and setbacks while ensuring inclusivity, respect for not only our populations but also in our field, and equity. We close with a proverb that we hope all attendees will follow: “*O ka pono ke hana ia a iho mai na lani*” [1], meaning “*continue to do good until the heavens come down to you*”, or “*blessings come to those who persist in doing good*.”

## Figures and Tables

**Table 1.** Correspondence between either Olelo Noeau and analogous ethical considerations in biomedical informatics research.

Olelo Noeau	English interpretation	Relevant Hawaiian concepts, values	Analogue in Biomedical Informatics
Ike aku, ike mai, kokua aku kokua mai; pela iho la ka nohona ohana	Recognize and be recognized, help and be helped; such is family life.	Ohana, Laulima	Inclusiveness, Human Centered Design Utilitarian ethics, Collaboration
Ike i ke au nui me ke au iki	Know the big current and the little current	Pono	Equity, Fairness
Kanukanu, huna i ka meheu, i ka maawe alanui o Kapuukolu	Covering with earth, hiding the footprints on the narrow trail of Kapuukolu	Kapu	Respect for privacy and sanctity
He waiwai nui ka lokahi	Unity is a precious possession	Lokahi	Balance of traditional performance metrics, privacy, and fairness
